# Neuronal Features of Visual Attention in the Mouse Superior Colliculus Depend on Learned Behavioral Relevance

**DOI:** 10.1523/JNEUROSCI.1187-25.2025

**Published:** 2026-01-07

**Authors:** Lupeng Wang, Christian Quaia, Kerry Elliott, Kara K. Cover, Richard J. Krauzlis

**Affiliations:** ^1^Laboratory of Sensorimotor Research, National Eye Institute, Bethesda, Maryland 20892; ^2^Laboratory of Neurogenetics, National Institute on Aging, Bethesda, Maryland 20892

**Keywords:** attention, learning, mouse, neuron, superior colliculus, visual

## Abstract

Visual selective attention is not wired into the brain fully formed and immutable but is acquired and refined as animals learn by interacting with their environment. Here we investigated this process by studying neuronal activity in the superior colliculus (SC) of male and female mice that learned different meanings of the same visual stimuli. We recorded spiking activity of neurons across the superficial and deep SC layers in three cohorts of mice, each trained using a tailored set of task rules that attributed different levels of behavioral relevance to the visual stimuli. Experimental conditions across the three tasks were carefully matched for visual stimulation and task engagement. We found that several markers of attention-related activity depended on the level of learned behavioral relevance, with the strongest effects in deep SC. First, for activity evoked by cue onset, visual responses in superficial SC were larger in animals that exhibited higher learned relevance. In deep SC, the presence of visual onset responses depended entirely on the learned behavioral relevance. Second, traditional attention-related modulation, defined as enhanced steady-state activity for the visual stimulus at the cued location, was also stronger with higher learned relevance, and this effect was limited to deep SC. Finally, the response to the visual change at the cued location was virtually absent when learned behavioral relevance was low but was prominent after visual training. Together, these results reveal that several fundamental features of attention-related modulation depend on the behavioral relevance learned through task-specific training, including aspects of stimulus-driven attention.

## Significance Statement

Selective attention requires the recruitment of circuits that are anatomically available in naive animals but that acquire specific functional roles with experience and training. This crucial aspect of attention is not yet well understood, because most studies of visual attention rely on well-trained animals. Here, by comparing SC activity across mice trained on tasks with distinct behavioral demands, we show that several fundamental neuronal signatures of visual attention depend on the learned behavioral relevance of visual stimuli, including aspects of stimulus-driven attention that are usually considered innate. Understanding how attentional circuits are shaped by experience can provide crucial insights into the functional logic of attention and reveal possibilities for how mechanisms of plasticity might address attention disorders.

## Introduction

The ability to efficiently process relevant visual stimuli is a core function of the primate brain, and much of what we know about this function and its underlying neural basis comes from non-human primates well-trained on visual attention tasks. Nonhuman primates are an important animal model because their visual system shares the same essential features with humans, notably foveal vision and a dominant visual pathway through the lateral geniculate nucleus. Studies in nonhuman primates have identified brain regions that play central roles in the control of selective attention, including the visual cortex (Reynolds and Chelazzi, 2004; Maunsell, 2015), frontal and parietal cortex (Bisley and Goldberg, 2010; Squire et al., 2013), thalamic nuclei (McAlonan et al., 2008; Saalmann and Kastner, 2011), the superior colliculus (Krauzlis et al., 2013), and basal ganglia (Gottlieb et al., 2014; Arcizet and Krauzlis, 2018). Nonhuman primates can perform complex attention tasks, enabling researchers to investigate specific attentional mechanisms, such as the role of correlated variability (Nienborg et al., 2012) and the distinction between changes in sensitivity and shifts in decision criteria (Luo and Maunsell, 2015).

Another important animal model for studying attention is the rodent, despite having a very different visual system (Huberman and Niell, 2011). Rodents have been used over many years to investigate sustained attention and behavioral vigilance (Bushnell, 1995), and it is now established that mice can use spatial cueing to improve their visual perceptual performance (Wang and Krauzlis, 2018), using paradigms adapted from classic tests of selective attention. In freely moving mice, spatial cues can likewise improve visual discrimination performance in a version of the well-known visual flanker task (You and Mysore, 2020).

The brain structures in the mouse associated with visual attention include the visual cortex (Speed et al., 2020), frontal cortex (Zhang et al., 2014), thalamus (Ahrens et al., 2015), superior colliculus (Wang et al., 2022), and basal ganglia (Wang et al., 2018). Studies of these structures have illustrated how the thalamic reticular nucleus gates the transmission of visual signals to the cortex (Wimmer et al., 2015), how local circuits in visual cortex are influenced by inputs from cingulate frontal cortex (Zhang et al., 2014), and how the direct pathway through the basal ganglia implements biases for spatial expectation during visual attention tasks (Wang and Krauzlis, 2020). The full impact of these mouse studies in understanding the circuits for attention in primates remains uncertain, but the genetic tools available in the mouse make it possible to study visual circuits (Seabrook et al., 2017) at a level of cellular detail that is not yet possible in nonhuman primates.

The strategy in most attention studies is to use well-trained subjects to get reliable and consistent performance. However, it is now generally recognized that the properties of visual circuits change with training (Karmarkar and Dan, 2006). For example, as mice are trained to perform a visual orientation discrimination task, the activity of neurons in visual cortex exhibit increased discriminability for the task-relevant stimuli (Jurjut et al., 2017), a signature of perceptual learning. How such changes might relate to selective attention is not known, but they likely bear on how visual circuits get recruited and modified to accomplish visual attention and possibly reveal how mechanisms of plasticity can be used to address disorders of attention.

Here we investigated this issue by building on our previous findings that the mouse superior colliculus (SC) is crucial for the performance of visual attention tasks (Wang et al., 2020) and that neurons in the SC of well-trained mice show attention-related modulation with spatial cueing (Wang et al., 2022). By recording neuronal activity in three cohorts of mice trained to attribute different levels of behavioral relevance to visual stimuli, under conditions matched for visual stimulation and task engagement, we found that several important features of attention-related modulation depend on task-specific learning, including aspects of stimulus-driven attention that are often viewed as fixed and automatic responses to salient visual events.

## Materials and Methods

### Animals

We used nine male and seven female wild-type C57BL/6J mice (JAX stock # 000664, Jackson Laboratory) housed in a reversed day–night cycle. All mice were in group housing (2–4 cage mates) prior to surgery and then singly housed after surgery and used in experimental procedures for up to ∼9 months. All experimental procedures and animal husbandry were approved by the National Eye Institute Animal Care and Use Committee (ACUC) and complied with Public Health Service policy on the humane care and use of laboratory animals.

### Surgical procedures

**Figure 1.**
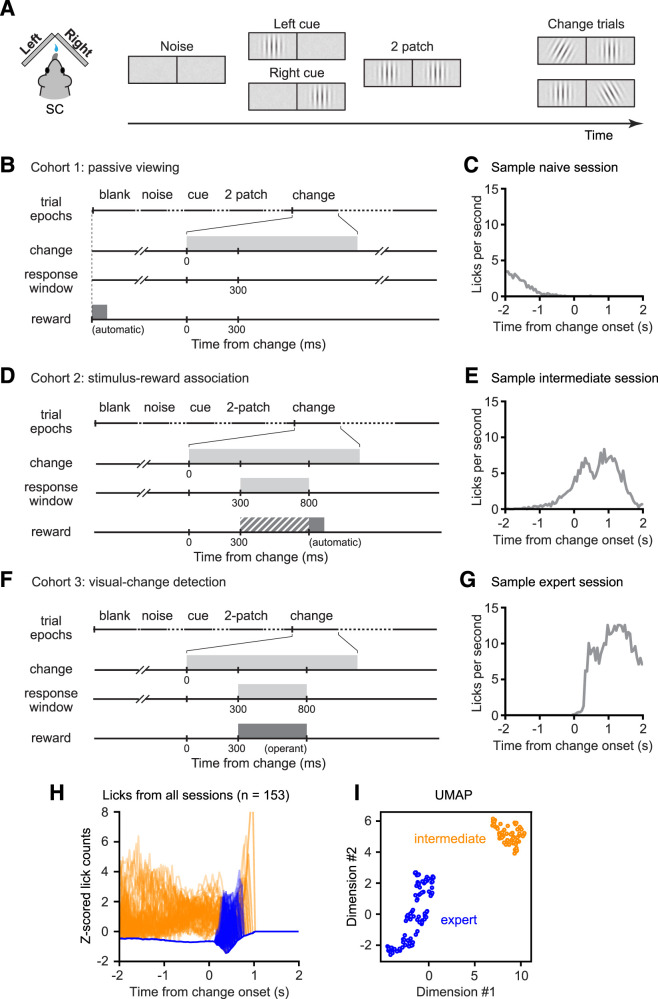
Behavioral approach for probing task-specific learning. ***A***, Schematic of the visual task. Left, Top-down diagram view of the mouse and visual displays. Right, Illustration of the sequence of visual stimuli presented on the left and right displays during each trial of the behavioral tasks. Trials with left or right cues were presented in interleaved blocks and the cue side predicted which stimulus could change. The same stimulus sequences were presented to all the mice. ***B***, The sequence of events in the “passive viewing” version of the task. A reward was delivered at the beginning of each trial, regardless of the licking behavior. ***C***, Histogram of lick events from a sample session of a mouse in the passive viewing condition. The frequency of licks is highest at the beginning of the trial. ***D***, Sequence of events in the “stimulus-reward association” version of the task. A reward was delivered shortly after the orientation change (filled bar in the reward trace) or immediately after licks that occurred within the response window (cross-hatched bar), whichever occurred first. ***E***, Histogram of licks from a sample session of a mouse in the stimulus-reward association task, identified as an “intermediate” session. The frequency of licks increases steadily during the trials and peaks after the orientation change event as the reward is delivered. ***F***, Sequence of events in the “visual-change detection” version of the task. Mice were rewarded only for licks that occurred within the response window and received a timeout penalty for anticipatory licks. ***G***, Histogram of licks from a sample session in the visual-change detection task, identified as an “expert” session. Licks start abruptly after change onset with minimal anticipatory licks. ***H***, Lick frequencies over time observed from all sessions in the stimulus-reward association and visual-change detection versions of the task. Colors are assigned based on how the lick values were segregated into intermediate (orange) and expert (blue) clusters using the Uniform Manifold Approximation and Projection (UMAP) method. The inputs to the UMAP were the traces shown here. ***I***, The first two dimensions of the UMAP method provided a robust segregation of the lick patterns into two distinct clusters—intermediate (orange) and expert (blue).

Mice were implanted with a head-holder at age 6–8 weeks, using methods described in detail previously (Wang et al., 2022). After recovering from the implant surgery, nine of the mice were trained on “stimulus-reward association” and “visual-change detection” versions of the visual task (Fig. 1) for 20–30 d before a 16-channel microwire recording bundle (Innovative Neurophysiology) was implanted in a second surgery. The bundle implantation took place after the behavioral assignment into intermediate and expert cohorts had been determined (i.e., expert cohort mice already showed expert performance). The remaining seven mice were trained on the “passive viewing” visual task, and the microwire recording bundle was implanted in the same surgery as the head-holder. Thus, for all three cohorts of mice, the neuronal recordings that followed were performed at similar times relative to the probe implantation. The microwire bundles were aimed at the superior colliculus, using a stainless steel guide tube and coordinates of ±0.8–1.1 mm from midline (M-L axis), −3.65–−4.0 mm from bregma (A-P axis) and 0.2–0.5 mm ventral (D-V axis, tip of the guide tube placed very shallow in cortex), referencing a standard mouse brain atlas (Paxinos and Franklin, 2019). The tip of the microwire bundle was therefore placed in cortex several millimeters above the SC at the time of surgical implantation. Mice received postsurgical analgesics (ketoprofen, 1.85 mg/kg) to alleviate potential discomfort.

### Food control

Mice were placed on a food control schedule after they recovered from surgery and returned to at least 95% of their presurgery weight (typically within 7–9 d). While on food control, their intake of dry food was controlled, but they had *ad libitum* access to water and they could augment their dietary intake by access to a nutritionally complete 8% soy-based infant formula (Similac). Overall food intake was regulated so that each mouse maintained at least 85% of their free-feeding body weight; the health status of each mouse was monitored daily throughout the study.

### Behavioral apparatus

Mice were first acclimatized to handling procedures by having their heads gently restrained while receiving manually delivered soy-based fluid through a sipper tube. Once mice were adapted to head restraint and the sipper tube, we switched to automatic delivery of fluid under computer control in the behavioral apparatus.

The behavioral apparatus has been described in detail previously (Wang and Krauzlis, 2018; Krauzlis et al., 2020). Briefly, it consisted of a custom-built enclosure that displayed visual stimuli to the mouse while they were head-fixed and positioned atop a polystyrene wheel. The mouse faced two visual displays, centered on axes 45° to the left and right of straight ahead, and the visual content of the displays was updated based on the rotation of the wheel as the mouse ran or walked along a virtual linear path. A reward delivery spout was positioned near the snout of the mouse, and lick contacts with the spout were detected by a piezo sensor using custom electronics. When lick contact was detected at appropriate times during the tasks, a small volume (5–10 μl) of an 8% solution of soy-based infant formula (Similac) was delivered through the spout by a peristaltic pump (Instech P720, Instech Laboratories) under computer control using a Datapixx device (Vpixx Technologies) and the Psychophysics Toolbox extensions (Brainard, 1997; Pelli, 1997) for Matlab (The MathWorks), controlled by Matlab-based routines run on a Mac Pro (Apple).

### Visual tasks

The tasks were based on those we previously used to manipulate visual spatial attention (Wang and Krauzlis, 2018). Experimental sessions were organized as blocks of randomly shuffled, interleaved trials, and each trial consisted of a sequence of epochs that the mouse progressed through by walking or running forwards on the wheel, with the epoch duration determined by the time required for the mouse to travel a randomized distance on the wheel. A typical trial lasted several seconds, and each mouse typically performed several hundred trials in each daily experiment.

In these experiments, each trial included a sequence of four epochs defined by the stimuli presented on the visual displays. In the first epoch (Fig. 1*A*, noise), the uniform gray of the intertrial interval was replaced by pink noise with an RMS contrast of 3.3%; this epoch was presented for a wheel distance of 10–20 cm (range of time, 0.2–0.3 s). In the second epoch (“cue”), a vertically oriented Gabor patch was added to the pink noise, centered in either the left or right visual display. The Gabor patch consisted of a sinusoidal grating (95% Michelson contrast) with a spatial frequency of 0.1 cycles per degree, modulated by a Gaussian envelope with full-width at half-maximum of 18° (*σ* = 7.5°). The phase of the grating incremented in proportion to the wheel rotation with every monitor refresh, so that the sinusoidal pattern was translated within the patch by approximately the same distance that the mouse traveled on the wheel, consistent with optic flow during locomotion. This second epoch lasted for 46–92 cm (0.36–1.55 s). In the third epoch (“2-patch”), a second vertically oriented Gabor patch appeared on the other visual display. This second Gabor stimulus had the same visual properties as the first, except that it drifted in the opposite direction. This epoch lasted for 107–214 cm (0.84–3.6 s). In the fourth epoch (“change”), the cued Gabor patch (i.e., the one that appeared first) changed its orientation. The amplitude of the orientation change was 9, 12, 15, 20, or 30°, selected to achieve hit rates of ∼80%. If the mouse licked within the response window after the orientation change, the response was scored a “hit” for that trial and the mouse received a fluid reward. To discourage guessing, on an equal number of trials the cued Gabor patch did not change its orientation at the onset of the fourth epoch (“no-change,” not illustrated). If the mouse correctly withheld from licking throughout the entire “change” epoch, the trial was scored as a “correct reject,” and the duration of the trial was extended to include an additional safety-net epoch during which the cued Gabor patch underwent a 30° orientation change and the mouse could receive a reward by licking within a comparable response window. As verified in previous studies (Wang et al., 2018; Wang and Krauzlis, 2018, 2020), this strategy successfully minimizes guessing behavior and keeps the task rule consistent for the mice to learn. Note that the “no-change” trials were not included in the data analysis. The average luminance across each visual display in all epochs was 4–8 cd/m^2^.

### Electrophysiological recording

Electrophysiological signals from the microwire bundles were processed using an RZ5D processor and the Synapse Suite interface (Tucker-Davis Technologies) with voltages bandpass filtered (0.3–7 kHz) and sampled at 25 kHz. The bundles were lowered along the dorsal-ventral axis with a microdrive included as part of the bundle assembly. Across each successive recording session, the wire bundle was advanced 100–150 μm to isolate activity from additional units at different depths. Spike clusters were sorted offline using KiloSort2. Clusters were then inspected by a human expert and manually curated based on spike waveforms, autocorrelograms, and cross-correlograms with neighboring clusters using Phy2. Single-unit clusters were vetted using in-house tools (https://github.com/ElKatz/kilo2Tools), based on waveform shape, stability throughout the session, peaked inter-spike interval distributions, and an interspike interval violation of <0.5% (defined by a refractory period of 1.25 ms).

**Figure 2.**
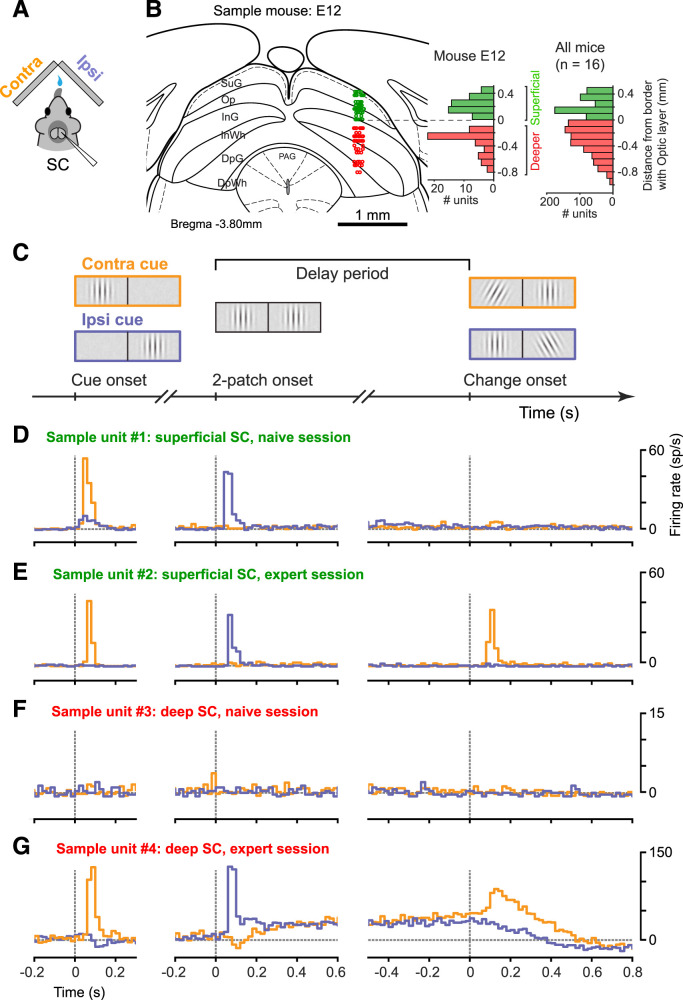
Neuronal activity varied with different levels of task performance. ***A***, Schematic of unilateral recording in the mouse SC during the visual tasks with stimuli placed contralateral (contra) and ipsilateral (ipsi) to the recording site. ***B***, Distribution of recording sites in the SC. Recording sites from one sample mouse are overlaid on a representative coronal anatomical section of the SC (left-most), indicating locations in the superficial (green) and deep (red) layers of the SC. The histograms indicate the numbers of units recorded at different depths within the SC for this sample mouse (left histogram) and pooled across all mice (*n* = 16). For pooling recording sites across mice (right histogram), the border with the optic layer (Op) is defined as zero mm. ***C***, Schematic of the visual task, indicating the principal visual events that anchor the analysis of the SC neuronal activity, especially cue onset and change onset. ***D***, Peri-event time histograms of firing rate for a sample neuron recorded in the SC superficial layer during a naive behavioral session. Data are aligned on cue onset (left), 2-patch onset (middle), and change onset (right). The data from trials with contralateral cues (orange) and ipsilateral cues (blue) are plotted separately. ***E***, Same as ***D***, but for a sample neuron recording in the superficial SC during an expert session. ***F***, Same as ***D***, but for a sample neuron recording in the deep SC during a naive session. ***G***, Same as ***D***, but for a sample neuron recording in the deep SC during an expert session.

Neurons were recorded across all depths of the SC (Fig. 2*B*). To determine the SC surface, mice were shown bilateral full-field drifting vertical sinusoidal gratings (∼0.1 cpd) as the probe was gradually lowered in 50 µm steps. The SC surface was defined as the first depth at which multiunit activity from at least one channel was modulated by the drifting visual grating. The bundle tracks for each mouse were reconstructed by identifying the gliosis in postmortem Nissl-stained tissue. Each relevant brain section was aligned on the standard mouse brain atlas (Paxinos and Franklin, 2019), and the depth in the SC of each recorded unit was then estimated based on the calibrated distance along the bundle track and referenced to the border between the stratum opticum and the intermediate gray. Neurons recorded above the stratum opticum (typically within the initial 400 μm from the surface) were considered to be within the superficial SC. Neurons below the stratum opticum but not deeper than 2 mm below the surface were considered to be within the intermediate and deep layers of the SC and analyzed together as one group (deep SC). A total of 1,587 units were judged to be well-isolated single neurons recorded in the SC (495 superficial layers, 1,092 deep layers) and were included in the data reported here.

### Monitoring mouse eye movements and pupil size

To examine the possible influence of eye movements on our neuronal data, we monitored eye position and pupil size using a 240 Hz CCD camera (ISCAN) during the entirety of the electrophysiology experiments. We imaged an area of 1.5 mm × 3 mm with a macro lens (ISCAN) centered on the eye. Four infrared light-emitting diodes (wavelength 940 nm) were used to illuminate the eye. Commercially available acquisition software (ETL-200, ISCAN) was used to determine the center and boundary of the pupil. Estimates of relative eye position were obtained by subtracting the center of corneal reflection from the pupil center to compensate any translational movement of the eye in the imaging plane due to head movements. Epochs of the eye movement data containing saccades were identified based on speed (>50°/s), amplitude (>2°), and duration (longer than 15 ms in duration). We quantified the frequencies of saccadic eye movements over time for the different cohorts of mice, aligned on the same task events as in the analysis of neuronal data.

### Experimental design and statistical analysis

Both male and female mice were used, and we did not observe any systematic difference in behavioral performance between sexes in this study. Firing rates of individual neurons are represented as peristimulus time histograms (PSTHs), using 20 ms nonoverlapping bins aligned to the onset of task epochs. The time course of each neuron's modulation was computed from spike counts in nonoverlapping 20 ms bins (aligned on specific epochs). To quantify the modulation of each unit for visual task events, we measured the area under the receiver operating characteristic curve (AROC) based on the spike counts taken from appropriately chosen time bins and conditions, as follows. To measure the “cue onset discriminability,” we compared firing rates in a baseline epoch (−100 to 0 ms before cue onset) to firing rates in a response epoch (40–140 ms after cue onset). To measure the “spatial cue discriminability,” we compared firing rates at the end of the delay period (−200 to 0 ms before change onset) between trials with contralateral versus ipsilateral cues (i.e., trials in which the initial Gabor patch appeared in the visual field contralateral versus ipsilateral to the recording site in the SC). To measure the “change onset discriminability,” we compared firing rates in a baseline epoch (−100 to 0 ms before change onset) to firing rates in a response epoch (40–140 ms after change onset).

Confidence intervals for the AROC of individual units and for the median value of the AROC across units were computed through bootstrapping (resampling with replacement, 1,000 times) and defined as the 2.5 and 97.5 percentiles of the distribution of resampled values. Significance of AROC results for individual units was assessed by the 95% CI of the unit AROC not overlapping 0.5 (low bound above 0.5, or high bound below 0.5).

To compare the AROC results across task performance levels, we included all trials rather than subdividing based on the trial outcome, because “hits” and “misses” were only well defined for the expert sessions. Differences in neuronal discriminability (AROC values) across task performance levels were assessed using a linear mixed-effects model and a session-level permutation test. For each pairwise state comparison, we fitted a mixed model of the following form:

$$\mathrm{ARO}C_{ij}=\beta_{0}+\beta_{1}\mathrm{Leve}l_{ij}+u_{j}+\varepsilon_{ij},$$

where *i* indexes neurons and *j* indexes recording sessions. Level*_ij_* is a binary fixed effect indicating the task performance level, and *u_j_* is a random intercept accounting for shared variance among neurons recorded in the same session. The significance of group differences was evaluated from the fixed-effect coefficient *β*_1_. Because the distribution of AROC values can often be not normally distributed, we also conducted a nonparametric permutation test on session-averaged AROC values. The observed difference in median AROC between states was compared with a null distribution generated by randomly permuting Level labels 10,000 times (two-sided). The resulting *p* value reflects the fraction of permutations yielding a median difference at least as extreme as observed. The *p* values for these tests are reported in Table 1.

**Table 1.** Summary of statistical tests for the AROC analyses

Epoch	SC layer	Tilt size	Comparison	*p* value		Significant	Figure panel
				Linear mixed model	Permutation test		
Cue onset	Superficial	N/A	Naive versus Intermediate	0.641	0.736		3*C*
			Intermediate versus Expert	0.202	0.199		3*C*
			Naive versus Expert	0.085	0.090		3*C*
	Deep	N/A	Naive versus Intermediate	4.31 × 10^−14^	2.00 × 10^−4^	*	3*E*
			Intermediate versus Expert	0.046	0.213		3*E*
			Naive versus Expert	1.73 × 10^−19^	2.00 × 10^−4^	*	3*E*
Delay period	Superficial	N/A	Naive versus Intermediate	0.253	0.179		4*C*
			Intermediate versus Expert	0.035	0.084		4*C*
			Naive versus Expert	0.195	0.453		4*C*
	Deep	N/A	Naive versus Intermediate	0.019	6.00 × 10^−4^	*	4*E*
			Intermediate versus Expert	0.053	0.392		4*E*
			Naive versus Expert	2.33 × 10^−7^	2.00 × 10^−4^	*	4*E*
Change onset	Superficial	Large	Naive versus Intermediate	0.260	0.601		5*D*
		Medium	Naive versus Expert	0.005	0.016	*	5*D*
		Small	Naive versus Expert	0.301	0.307		5*D*
	Deep	Large	Naive versus Intermediate	1.35 × 10^−8^	2.00 × 10^−4^	*	5*E*
		Medium	Naive versus Expert	8.54 × 10^−6^	0.014	*	5*E*
		Small	Naive versus Expert	1.33 × 10^−8^	2.00 × 10^−4^	*	5*E*

**Figure 3.**
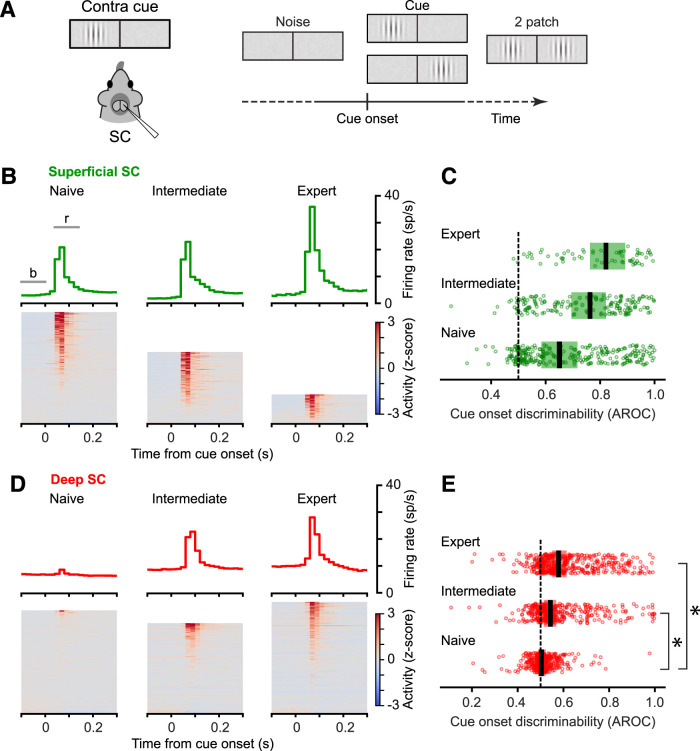
Visual responses to cue onset increased with task performance level. ***A***, Schematic illustrating the visual conditions at cue onset (left) and the timing of the cue onset in the trial sequence (right). ***B***, Visual responses to cue onset in the superficial SC in sessions with different levels of task performance. Population average firing rates are shown (top) as peri-event time histograms for superficial SC neurons recorded during naive (left, *n* = 261 neurons), intermediate (middle, *n* = 167), and expert (right, *n* = 67) sessions. The horizontal bars indicate the baseline (b) and response (r) intervals used for the AROC analyses in ***C*** and ***E***. The heat maps (below) illustrate the average firing rate for each superficial neuron separately. The data from each neuron are shown as one row in the heat map, sorted and ranked based on the size of their *z*-scored responses (response epoch minus baseline epoch), from highest (top row) to lowest (bottom row) activity. The heat maps differ in height across the three levels of task performance, reflecting the different numbers of neurons recorded. ***C***, Discriminability of the visual cue onset in the superficial SC increased with task performance level. Cue discriminability was measured from the area under the ROC curve (AROC) based on the firing rates in the response and baseline intervals illustrated in ***B***. The horizontal position of each green circle indicates the AROC value computed from one superficial SC neuron, grouped by performance level into separate bands, and vertical spaced just to reduce overlap of the symbols. The thick vertical black tics indicate the population means and the shaded regions represent the 95% confidence intervals. The dashed line at 0.5 indicates chance performance. Significant differences (*p* < 0.05) were determined using a linear mixed-effects model with random intercepts per session and a session-level permutation test (10,000 label shuffles, two-sided); comparisons that passed both tests for significance are indicated with asterisks. *p* values are reported in Table 1. ***D***, Visual responses to cue onset in the deep SC in sessions with different levels of task performance. Population average firing rates (red) are based on 369 (naive), 323 (intermediate) and 400 (expert) neurons. Other conventions same as in panel ***B***. ***E***, Discriminability of the visual cue onset in the deep SC (red) increased with task performance level. Conventions same as in panel ***C***.

**Figure 4.**
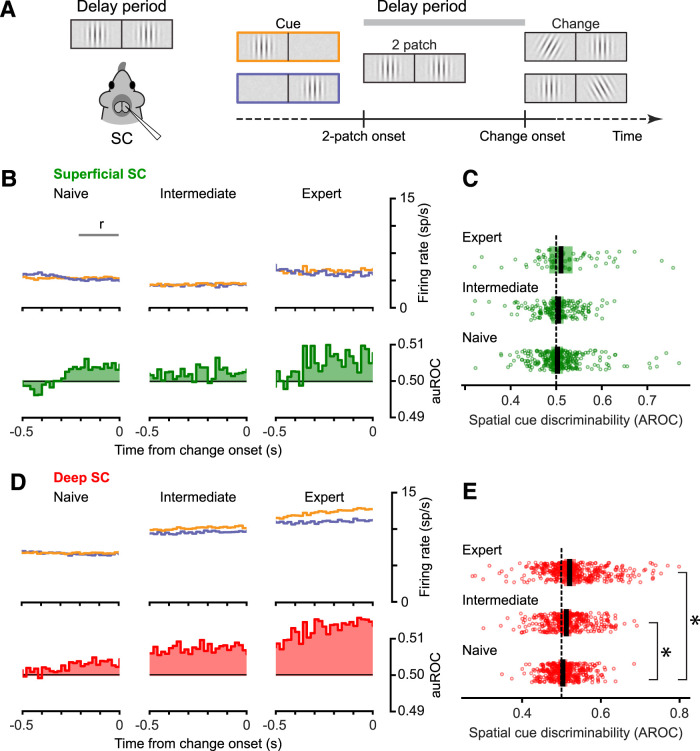
Spatial cue-related modulation in the SC increased with task performance level. ***A***, Schematic illustrating the visual conditions during the delay period (left) and the timing of the delay period with respect to the 2-patch and change onset. The analysis focused on the activity observed at the end of the delay period and is aligned to end with the onset of the orientation change. ***B***, Activity during the delay period in the superficial SC did not show substantial spatial cue-related modulation, regardless of the level of task performance. Population average firing rates are shown (top) for superficial SC neurons separately from trials with contralateral (orange) and ipsilateral (purple) spatial cues, recorded during naive (left, *n* = 261 neurons), intermediate (middle, *n* = 167), and expert (right, *n* = 67) sessions. The horizontal bar indicates the response (r) interval used for the AROC analyses in ***C*** and ***E***. The shaded histograms (below) indicate the average spatial cue discriminability across all neurons, with values above 0.5 indicating preference for the contralateral cue condition. ***C***, Spatial cue discriminability in the superficial SC did not increase substantially with task performance level. Spatial cue discriminability was measured from the area under the ROC curve (AROC) based on the firing rates in the contralateral and ipsilateral response intervals illustrated in ***B***. Significant differences were identified as in Figure 3*C*. *p* values are reported in Table 1. Other conventions are the same as in Figure 3*C*. ***D***, Activity during the delay period in the deep SC showed greater spatial cue-related modulation with increased task performance level. Population average firing rates for deep SC neurons in trials with contralateral (orange) and ipsilateral (purple?) spatial cues, recorded during naive (left, *n* = 369 neurons), intermediate (middle, *n* = 323), and expert (right, *n* = 400) sessions. Other conventions same as in panel ***B***. ***E***, Spatial cue discriminability in the deep SC (red) increased with task performance level. Other conventions same as in panel ***C***.

**Figure 5.**
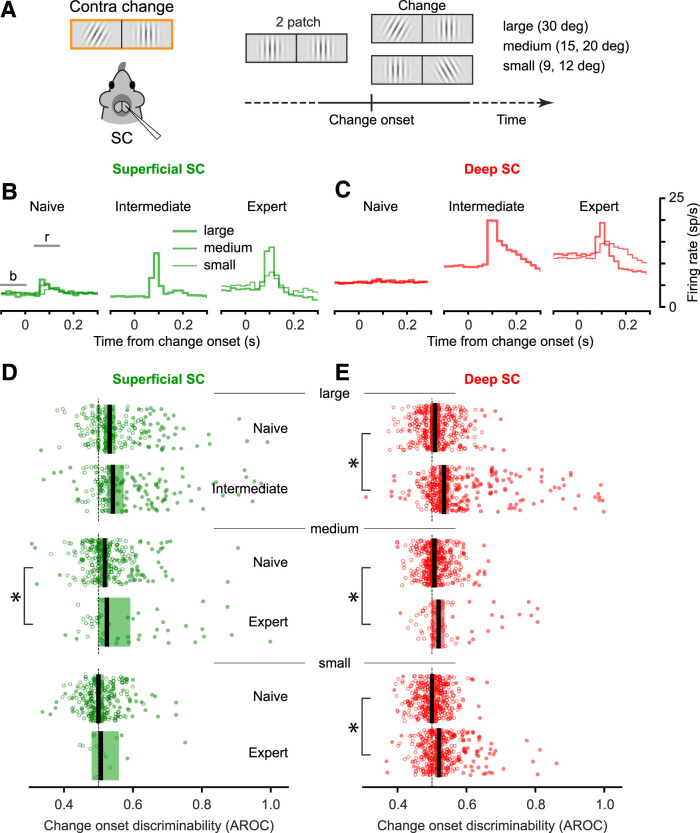
Change-related responses in the SC increased with task performance level. ***A***, Schematic illustrating the visual conditions at change onset (left) and the timing of the change onset in the trial sequence (right). Across the set of experiments, we tested five different orientation change amplitudes, which we categorized as large (30°), medium (15 and 20°), and small (9 and 12°). ***B***, Visual responses to the orientation change onset in the superficial SC in sessions with different levels of task performance. The different line thicknesses show data from different orientation change amplitudes. ***C***, Visual responses to the orientation change onset in the deep SC in sessions with different levels of task performance. Other conventions are the same as in Figure 3*B*. ***D***, Discriminability of the orientation change onset in the superficial SC increased with task performance. Change onset discriminability was measured from the area under the ROC curve (AROC) based on the firing rates in the response and baseline intervals illustrated in ***B***. The horizontal position of each green circle indicates the AROC value computed from one superficial SC neurons, grouped by task performance level into separate bands, and vertical spaced just to reduce overlap of the symbols. The thick vertical black tics indicate the population means and the shaded regions represent the 95% confidence intervals. Significant differences were identified as in Figures 3 and 4. *p* values are reported in Table 1. ***E***, Discriminability of the orientation change onset in the deep SC (red) increased with task performance level. Conventions same as in panel ***D***.

Results from each of the statistical tests performed comparing the AROC results across task performance levels. Values are organized by the epoch analyzed (cue onset, delay period, change onset), and correspond to the data presented in Figures 3–5.

### Data availability

All the data were acquired and initially processed using custom scripts. The data analysis code and data that support the findings of this study will be made available from the corresponding author upon reasonable request.

## Results

### Behavioral approach for probing task-specific learning

To investigate how neuronal activity in the SC depends on visual learning, we studied three cohorts of mice (*n* = 17) that were trained to perform three different but closely related behavioral tasks, under conditions carefully matched for visual stimulation and level of task engagement. The primary task was our previously described visual attention task (Wang and Krauzlis, 2018), in which mice were trained to quickly detect a change in the orientation of a visual stimulus to receive their reward. In addition, we included two versions in which we simplified the reward contingencies to reduce the behavioral relevance of the visual stimuli and especially the visual change event. In all three tasks, the mice experienced the same sequence of visual stimuli but the differences in reward structure led to them expressing different beliefs about the behavioral relevance of visual stimuli and visual events, as evident in their patterns of licking. We first briefly describe the sequence of visual stimuli, which were the same as those used previously (Wang et al., 2022), and then explain the differences across the three tasks.

Visual stimuli were presented on a pair of lateralized displays to head-fixed mice as they walked or ran on a wheel. The rotation of the wheel controlled each trial's progression through several distinct visual epochs (Fig. 1*A*) and determined the drift rate of Gabor stimuli, consistent with movement down a virtual corridor. After the start of each trial, pink noise was presented on both displays (“Noise”), followed by presentation of a single lateralized vertical Gabor patch. This Gabor patch served as a spatial cue, indicating the location of the possible upcoming orientation change (“Left cue,” “Right cue”). The appearance of a second Gabor patch in the other visual hemifield marked the start of the “2 patch” epoch. Finally, on a random half of the trials the Gabor patch on the cued side changed its orientation (“Change”); on the other half of the trials, both patches remained vertical in a seamless extension of the 2-patch epoch (“No change,” not shown and not included in the data analysis).

This identical sequence of visual stimuli was presented to mice in each of the three training cohorts, but the mice were trained to different levels of task performance based on the duration of the training they received and the details of the visual-based contingencies in the task. Specifically, the training tasks differed in two key aspects: the timing of the reward delivery with respect to the sequence of visual epochs and whether the reward delivery was contingent on licking at the time of the orientation change. Aside from these task differences, all three cohorts were on the same food control schedule, performed the same locomotor behavior to progress through the task epochs, and performed similar numbers of trials in their daily sessions.

The first cohort of mice (*n* = 7) was trained on a “passive viewing” version of the task (Fig. 1*B*). The soymilk reward was automatically delivered during the blank inter-trial interval preceding the noise epoch; thus, the subsequently viewed visual epochs were never linked to reward timing. Consequently, these mice mostly licked early in the trial and made very few licks by the time of the orientation change (Fig. 1*C*). The nominal hit rates for these mice (i.e., based on licks within the 300–800 ms window following the orientation change) were very low (mean across sessions: 0.025; bootstrap 95% CI: [0.017–0.033]).

The second cohort of mice (*n* = 6) was trained on a “stimulus-reward association” version of the task (Fig. 1*D*). The reward was delivered 800 ms following the orientation change, providing an opportunity for the mice to associate the presence of the Gabor patches and the orientation change with the reward. These mice also received rewards immediately after licks that occurred within the response window following (300–800 ms) the orientation change. To reinforce the association between the change event and reward delivery, this cohort of mice was not exposed to no-change catch trials. Mice in this cohort tended to display steadily increasing rates of licking during each trial, continuing through the time of the orientation change (Fig. 1*E*), consistent with having learned something about the relevance of the visual stimuli and the temporal structure of the task. These mice had hit rates that, across sessions, had a mean value of 0.666 (95% CI: 0.588–0.739).

The third cohort of mice underwent further operant training until they became experts on the “visual-change detection” task. These mice received no automatic rewards but were required to lick within the response window following the orientation change (Fig. 1*F*). Moreover, they were required to make no anticipatory licks before the orientation change, otherwise the trial was aborted, and they were subject to a timeout penalty. Mice that achieved expert levels of performance (*n* = 4) had average hit rates of 0.832 (95% CI: 0.816–0.848) during recording sessions, and their licks were sharply time-locked to the orientation change (Fig. 1*G*).

### Classifying the level of visual task performance on individual recording sessions

We commenced neuronal recordings in the SC after mice in each of the three cohorts were trained to an appropriate level of task performance. For the mice in the passive viewing cohort, this began soon after mice were acclimatized to the apparatus, and the task performance level in all these recording sessions was defined as visually “naive.” For the mice in the other two cohorts, we expected that the level of task performance exhibited during each recording session would largely be determined by the training paradigm, but rather than assuming this to be the case, we classified the session-by-session learning status of each mouse, based on the pattern of licks during each session. Across all individual neuronal recording sessions, the lick counts over time displayed variable but distinctive patterns (Fig. 1*H*) that we identified using a Uniform Manifold Approximation and Projection (UMAP) method. Using the first two UMAP dimensions, we objectively classified the level of task performance demonstrated during each recording session of these mice as “intermediate” or “expert” (Fig. 1*I*). These session-by-session task-level classifications closely matched the training cohort distinctions (259/265 sessions) and provided the basis for grouping the neuronal recording data described in the subsequent sections. The slight discrepancy between the session-by-session classifications and the training cohorts is explained by one mouse that transitioned from intermediate to expert levels of performance during the course of the recording sessions. Consistent with our previous behavioral results establishing this visual attention task in mice (Wang and Krauzlis, 2018), during sessions classified as “expert,” hit rates and sensitivity (measured as *d*’) were slightly but significantly higher on trials that included the spatial cue (as described above) compared with probe trials in which this spatial cue was omitted (hit rates: 85% vs 77.8%, *p* = 0.009; sensitivity: 1.66 vs 1.47, *p* = 0.039; reaction times: 450 ms vs 462 ms, *p* = 0.032; all Wilcoxon signed-rank tests).

### Neuronal activity was markedly different with different levels of visual task performance

Neuronal recordings were obtained using chronically implanted 16-channel moveable microwire bundles that were advanced through the SC superficial and deeper layers of 17 mice. From a total of 265 recording sessions, we recorded and analyzed the activity of 1,587 well-isolated neurons located within the SC (Fig. 2*A*,*B*).

We found that SC neuronal activity during the visual task (Fig. 2*C*) was strongly dependent on the level of visual task performance, in both the superficial and deep layers of the SC. Some of the most salient differences can be illustrated with a few sample neurons. In the superficial SC, there were brisk responses to the onset of the Gabor stimulus when it appeared in the contralateral visual field regardless of performance level. In contrast, responses to the orientation change were largely absent in naive sessions (Fig. 2*D*) but emerged in expert sessions (Fig. 2*E*); otherwise, there was very little task-related modulation. In the deep SC, there was little task-related modulation overall in naive sessions (Fig. 2*F*), but during expert sessions there were prominent visual responses to the orientation change as well as at stimulus onset and also tonic modulation during the delay period (Fig. 2*G*).

To document these main effects across our dataset, we focused our analyses on three task epochs: at cue stimulus onset, to assess the level of visual responsiveness; during the delay period, to test how spatial cueing affected SC activity; and at the orientation change, to measure the activity evoked by the visual event that acquired behavioral relevance through training.

### Visual responses to cue onset were larger with more visual training

On trials with contralateral spatial cues, the first visual stimulus to appear on each trial was a Gabor patch in the response field (Fig. 3*A*). In the superficial SC, the onset of this contralateral stimulus evoked brisk visual responses, and the traces showing the population average firing rates aligned on cue onset (Fig. 3*B*, top) illustrate that the amplitude of this visual response was larger in sessions performed at intermediate and expert levels. As illustrated by the rows of the heatmaps showing data from individual neurons, the prevalence of this visual evoked activity also depended on performance level (Fig. 3*B*, bottom). To quantify these effects, we computed a “cue discriminability” for each neuron by comparing the firing rates in a response window (40–140 ms after cue onset) to the firing rates in a baseline interval (−100 to 0 ms before cue onset) using the area under the receiver operating characteristic curve (i.e., AROC values). This analysis confirmed that SC neurons positively discriminated the cue onset from the baseline (i.e., AROC values were >0.5), and this discriminability increased with the level of task performance (Fig. 3*C*; Median AROC [95% CI]: Naive: 0.650 [0.595–0.706]; Intermediate: 0.760 [0.698–0.811]; Expert: 0.826 [0.744–0.878]). Across our set of SC neurons, the prevalence of significant positive discriminability also increased with performance level (AROC significantly larger than 0.5: Naive: 65.1%; Intermediate: 79.0%; Expert: 94.0%). Conversely, the prevalence of significant negative discriminability decreased with performance level (AROC significantly smaller than 0.5: Naive: 2.7%; Intermediate: 1.2%; Expert: 0.0%).

In the deep SC, the onset of the cue stimulus evoked a negligible visual response in naive sessions, but substantial visual responses in intermediate and expert sessions (Fig. 3*D*, top). As illustrated by the heatmaps showing data from individual neurons, the fraction of neurons that exhibited a noticeable visual response also markedly increased in intermediate and expert recording sessions (Fig. 3*D*, bottom). These impressions were confirmed by our analysis of neuronal discriminability—the population of deep SC neurons did not discriminate the cue onset in naive sessions but did significantly discriminate the cue in intermediate and expert sessions (Fig. 3*E*; Median AROC [95% CI]: Naive: 0.504 [0.502–0.508]; Intermediate: 0.542 [0.527–0.555]; Expert: 0.578 [0.565–0.601]), and the prevalence of neurons with significant positive discriminability also increased (AROC significantly larger than 0.5: Naive: 17.6%; Intermediate: 44.9%; Expert: 55.5%). Conversely, the prevalence of significant negative discriminability decreased (AROC significantly smaller than 0.5: Naive: 6.2%; Intermediate: 7.7%; Expert: 2.8%). Direct comparisons of the AROC values across the three cohorts showed significant increases in cue onset discriminability with performance level (Fig. 3*E*).

### Spatial cue-related modulation in deep SC increased with more visual training

The onset of the second Gabor patch marked the beginning of the delay period in the visual task, which lasted until the cued patch might change its orientation (Fig. 4*A*). During the delay period, the two Gabor patches provided identical visual stimulation, but the preceding visual cue and the blocking of trials provided information about which patch might undergo an orientation change. We previously demonstrated that this spatial cueing could support attention-related improvements in task performance in mice (Wang and Krauzlis, 2018) and that it also produces spatial cue-related modulation in SC neuronal activity in expert mice (Wang et al., 2022). Here we report that this attention-related modulation varied with SC layer and level of visual task performance.

For superficial SC units, during naive and intermediate sessions the spike rates recorded showed only minor differences between contralateral versus ipsilateral spatial cue conditions (Fig. 4*B*, top left and middle panels). During expert sessions, there appeared to be a more consistent tendency for the firing rates to separate in favor of trials with contralateral spatial cues, starting ∼300–400 ms before the onset of orientation change (Fig. 4*B*, top right panel). To quantify this possible cue-related modulation, we computed “cue discriminability” (i.e., AROC values) for each neuron by comparing spike rates in ipsi-cue trials (“signal absent”) to spike rates in contra-cue trials (“signal present”) in consecutive nonoverlapping 20 ms bins. The average of these values across the population (Fig. 4*B*, bottom) suggests the presence of very slight cueing effects. However, overall, the cue discriminability was marginal for all three performance levels (Fig. 4*C*; Median AROC [95% CI]: Naive: 0.503 [0.496–0.508]; Intermediate: 0.505 [0.492–0.529]; Expert: 0.504 [0.501–0.507]), and the fraction of individual neurons with significant cueing effects was also small (AROC significantly larger than 0.5: Naive: 11.9%; Intermediate: 9.6%; Expert 17.9%; significantly smaller than 0.5: Naive: 4.6%; Intermediate: 8.4%; Expert: 6.0%). Comparisons of the AROC values for cue discriminability across the three cohorts did not show significant effects.

For deep SC units, the cue-related modulation was more evident and larger in sessions performed at intermediate and expert levels. The differences between the delay period firing rates in the contralateral and ipsilateral spatial cue conditions were negligible in naive sessions, but differences were more evident in intermediate and expert sessions, as evidenced by both the divergence in the population firing rates and the amplitude of cue discriminability (AROC values) over time (Fig. 4*D*). Across the population of neurons, cue discriminability significantly increased with performance level (Fig. 4*E*; Median AROC [95% CI]: Naive: 0.504 [0.501–0.507]; Intermediate: 0.513 [0.506–0.518]; Expert: 0.521 [0.514–0.529]) and so did the percentage of neurons with positive significant cue discriminability (from 11.4% in Naive to 17.0% in Intermediate to 23.3% in Expert). The percentage of neurons with negative cue discriminability was low overall (Naive: 4.6%; Intermediate: 7.1%; Expert: 4.3%). Comparisons of the AROC values across the three cohorts showed a significant increase in cue discriminability with higher task performance levels (Fig. 4*E*).

### Visual change-related activity required some level of task relevance

The onset of the change in the orientation of the Gabor patch (Fig. 5*A*) marked the availability of reward for mice in intermediate and expert sessions but was largely irrelevant for naive performance (Fig. 1). Consistent with this difference in behavioral relevance, in the superficial SC, the change in the orientation of the Gabor patch evoked negligible population activity during naive sessions, but substantial and more prevalent visual responses in the population activity in the intermediate and expert sessions (Fig. 5*B*). This increase in evoked activity was evident in the intermediate and expert mice, even though the amplitude of the orientation change was reduced from large (30°) in intermediate mice to medium (15 and 20°) and small (9 and 12°) in expert mice.

We found a similar pattern for the deep SC neurons. The stimulus tilt induced a robust visual response in the population activity during intermediate and expert, but not naive, sessions (Fig. 5*C*). This absence of change-related activity in naive sessions was observed regardless of whether the orientation change was small, medium, or large. In contrast, the population activity during intermediate sessions showed a robust modulation to the large orientation change, and during expert sessions there was strong modulation even for the medium and small changes.

Across the population of superficial SC neurons (Fig. 5*D*), the change-related discriminability (AROC values) was larger in intermediate than in naive mice for the large orientation changes (Median AROC [95% CI]: Naive: 0.533 [0.522–0.543]; Intermediate: 0.544 [0.531–0.567]) and larger in expert than in naive mice for the medium orientation change (Median AROC [95% CI]: Naive: 0.517 [0.509–0.523]; Expert: 0.531 [0.507–0.588]). The percentage of individual neurons with significant positive change-related discriminability (AROC significantly larger than 0.5) increased with performance level for large orientation changes (Naive: 47/204 = 23%; Intermediate: 80/167 = 47.9%) and for medium orientation changes (Naive: 45/255 = 17.6%; Expert: 23/52 = 44.2%). Comparisons of the AROC values across the cohorts showed a significant difference in change-onset discriminability between the naive and expert mice for the medium orientation change but not the other two conditions (Fig. 5*D*).

These differences were more pronounced among deep SC neurons (Fig. 5*E*). The change discriminability was larger in intermediate than in naive mice for the large orientation change (Median AROC [95% CI]: Naive: 0.508 [0.500–0.517]; Intermediate: 0.534 [0.526–0.544]), larger in expert than in naive mice for the medium orientation change (Median AROC [95% CI]: Naive: 0.507 [0.500–0.510]; Expert: 0.518 [0.505–0.531]), and larger in expert than in naive mice for the small orientation change (Median AROC [95% CI]: Naive: 0.500 [0.500–0.503]; Expert: 0.520 [0.515–0.525]). The percentage of individual neurons with significant positive change-related discriminability increased with performance level for large orientation changes (Naive: 13/340 = 3.8%; Intermediate: 120/323 = 37.2%), medium orientation changes (Naive: 22/369 = 6.0%; Expert: 16/71 = 22.5%), and small orientation changes (Naive: 7/369 = 1.9%; Expert: 52/329 = 15.8%). Comparisons of the AROC values across the cohorts showed a significant increase in change-onset discriminability with higher performance levels for all three orientation-change amplitudes (Fig. 5*E*).

### Probability of saccades decreased with higher task performance levels

**Figure 6.**
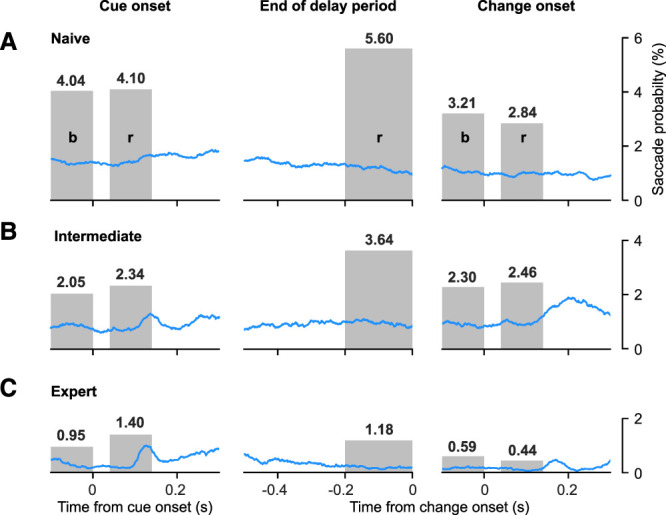
Probability of saccades decreased with task performance level. ***A***, Saccade probability as a function of time from naive sessions, aligned on cue onset (left), the delay period preceding the orientation change (middle) and the change onset (right). Gray bars and associated numbers indicate the saccade probability (%) in the baseline (b) and response (r) intervals, as described in Figures 3–5. ***B***, Saccade probability from intermediate sessions. ***C***, Saccade probability from expert sessions.

One possible explanation for the differences in task-related neuronal activity we observed with visual training is changes in eye movements. Specifically, given the role of the SC in the control of saccadic eye movements, the changes could be due to mice making more saccades time-locked to the occurrence of visual task events during expert and intermediate sessions than during naive sessions. To address this possibility, we quantified the probability of saccades aligned to the same task events as for the neuronal analyses (Fig. 6*A*). Indeed, we found small differences in saccade probability that depended on the level of task performance. However, in all time intervals saccades were more frequent in naive sessions (Fig. 6*B*) than in intermediate (Fig. 6*C*) and expert (Fig. 6*D*) sessions. Thus, the increases in phasic task-related activity we observed in intermediate and expert sessions cannot be explained by saccade-related increases in activity. Moreover, we repeated all the analyses based on trials without saccades in the relevant epoch and found that all the main effects were still present and significant, as might be expected given the overall low incidence of saccades.

## Discussion

By recording neuronal activity across the superficial and deep layers of the SC in mice trained on tasks designed to titrate the behavioral relevance of visual stimuli, we found that several basic features of attention-related modulation depend on task-specific learning, including aspects of stimulus-driven activity that might otherwise been viewed as pre-determined and automatic responses to salient visual events. In the superficial SC, the visual response evoked by the onset of the cue stimulus was present in naive sessions, as might be expected, but became strongly amplified with visual training. The phasic responses to the change in orientation of the Gabor patch—the relevant event in our attention task—were very small in naive sessions but became more prominent in intermediate and expert sessions. In the deep SC, there were barely detectable responses to the cue onset and the orientation change in naive sessions, but prominent responses to both stimulus events were evident in intermediate and expert sessions. In addition, the spatial cue-related modulation during the delay period—a traditional period for assessing attention-related modulation independent of visual stimulation—was absent in naive sessions but was a distinctive feature of activity from intermediate and especially expert sessions. Together, these results illustrate that selective attention involves the recruitment of circuits that acquire specific functional properties through experience and training. If the SC is viewed as a priority map, combining the influences of both top-down behavioral relevance and bottom-up salience to control target selection (Fecteau and Munoz, 2006) and visual attention (Krauzlis et al., 2013), then our results show that even the bottom-up salience component of this priority calculation is subject to learning driven by task demands.

### The SC and visual selective attention

The SC is an opportune place to look at how attention circuits change with experience, because it is a central node in attention control (Krauzlis et al., 2013; Basso and May, 2017). The earliest evidence for attention-related modulation of neuronal activity was obtained in the SC of nonhuman primates (Goldberg and Wurtz, 1972), and subsequent studies demonstrated that the primate SC plays a causal role in the control of visual spatial attention (Cavanaugh and Wurtz, 2004; Lovejoy and Krauzlis, 2010), even in tasks that exclude orienting movements and instead require covert shifts of attention (Herman et al., 2018). The role of the SC in selective attention is believed to be an extension of its evolutionarily conserved role in the selection of targets for orienting movements (Krauzlis et al., 2013); the homolog of the SC in non-mammalian species—the optic tectum—plays a key role in target selection and spatial attention (Knudsen, 2011, 2018). The primate SC is linked to the control of selective attention through several circuits (Krauzlis et al., 2013; Basso and May, 2017), and rather than exerting a major effect on extrastriate cortex (Zenon and Krauzlis, 2012), it instead targets an attention-related region in the ventral stream (Bogadhi et al., 2018, 2019) where it contributes to visual object processing as well as selective attention (Bogadhi et al., 2021).

Studies in mice have similarly demonstrated the importance of the SC for visual attention and target selection. The mouse SC is the primary recipient of retinal inputs (Ellis et al., 2016), and suppression of SC neuronal activity causes major impairments in visual task performance (Wang et al., 2020). Circuits within the mouse SC play crucial roles in interpreting those visual signals and transforming them, along with other signals, into appropriate behavioral responses (Cang et al., 2024). During manipulations of visual attention, neurons in the deeper layers of the mouse SC exhibit cue-related modulation and phasic responses to near-threshold visual events (Wang et al., 2022), demonstrating a bias in visual processing in favor of task-relevant spatial locations and stimulus events. Ascending circuits from the mouse SC through the thalamus target a set of higher visual areas that prefer object motion over self-motion (Beltramo and Scanziani, 2019; Brenner et al., 2023), suggesting a possible analog of the ascending attention-related SC circuits in the primate.

Our results reveal that many of these attention-related properties, often assumed to be fundamental properties of SC neurons, are not present in the naive state but require learning. The SC presumably acquires these properties through plastic changes of the circuits within and through the SC, as the mouse explores and learns about their environment. Depending on one’s view of attention, these acquired properties might also be viewed as changes in perceptual learning or spatial expectations, and we do not have a clear basis for distinguishing between these interpretations. Nonetheless, our results indicate that learning the visual task changed how SC neurons process specific sensory events, as distinct from nonspecific effects of motivation or arousal.

### Possible circuits underlying changes in SC neuronal properties

There are several possible circuits that could explain the differences we observed with visual training. One possibility are changes in the local circuitry within the SC (Isa and Hall, 2009). In the superficial layers, the larger phasic responses to visual events observed with training could be generated by facilitating the smaller transient response present in naive sessions, for example, by changes in inhibitory transmission (Kaneda et al., 2008). Such changes in local inhibition in superficial SC could potentially be mediated by cholinergic modulatory inputs to the superficial layers from the parabigeminal nucleus (Lee et al., 2001; Tokuoka et al., 2020). Other circuit mechanisms could increase the transmission of visual signals from the superficial layers to the deep layers of the SC (Phongphanphanee et al., 2008; Vokoun et al., 2010), potentially explaining the large effects we observed in the deep layers of the SC. Lateral interactions across the deeper layers of the SC could also support the competitive processing of visual signals (Phongphanphanee et al., 2014; Lintz et al., 2019; Essig et al., 2021), providing a possible explanation for the spatial cue-related modulation during our task.

Another possibility is that these properties are inherited from the cerebral cortex, especially the mouse primary visual cortex (V1). Activity in mouse V1 is necessary for normal performance on orientation change-detection tasks (Glickfeld et al., 2013), and selective ablation of visual cortical neurons that project to the SC increases detection thresholds (Ruediger and Scanziani, 2020). There is also ample evidence that visual processing in mouse V1 undergoes substantial changes as mice learn visual tasks. The representations of task-relevant features in mouse V1 improve and expand with training (Poort et al., 2015; Jurjut et al., 2017; Corbo et al., 2022), including not just changes in visual-evoked activity but also the emergence of anticipatory activity before expected visual events, analogous to the spatial cue modulation we observed during the delay period. There are multiple possible mechanisms for these changes in mouse visual cortex (Katzner et al., 2019), including circuit-level reorganization of functional connectivity across mouse V1 and cellular-level structural changes to synapses and neurons (Poort et al., 2022; Akitake et al., 2023; Lopez-Ortega et al., 2024).

Beyond visual cortex, areas of the mouse prefrontal cortex, especially the anterior cingulate cortex, are also potential candidates. Visual responsiveness emerges in the medial prefrontal cortex as mice are trained on visual tasks, and these changes are tightly linked to improvements in behavioral performance (Wal et al., 2021; Peters et al., 2022). The emergence of this cue-related modulation may be related to the role of the cingulate cortex in predicting outcomes from the actions performed in visual tasks (Akam et al., 2021). Conversely, suppression of activity in the anterior cingulate cortex increases errors in a visual discrimination task and these effects get larger with training (Weible et al., 2019). The mouse anterior cingulate cortex exerts strong attention-related effects on visual cortex through its direct feedback projections (Zhang et al., 2014), but it also has direct projections to the SC with functions that have not yet been clearly identified (Zhang et al., 2016).

Circuits through the basal ganglia are another possibility, especially those portions that route visual signals through the substantia nigra to the SC. In well-trained mice, activation of the “direct” pathway originating in the dorsomedial striatum shifts the decision criterion selectively when spatial attention is directed to the contralateral visual field (Wang et al., 2018; Wang and Krauzlis, 2020). These effects are consistent with reducing the tonic inhibition exerted on the SC from the substantia nigra (Hikosaka and Wurtz, 1985), which could explain the differences in SC activity during the delay period of our task and possibly the amplification of visual responsiveness. These circuits are likely recruited during training, since ablation of visual cortex neurons that project to the striatum reduces the speed of learning on visual tasks (Ruediger and Scanziani, 2020). In addition to signals from the visual cortex, these basal ganglia circuits process a wealth of task-related inputs, including from prefrontal cortex (Zhang et al., 2016) and the thalamus (Cover et al., 2023).

Teasing apart the contributions and interactions across these many brain regions is a complex problem but will provide important insights into how the full properties of visual selective attention are constructed by learning processes driven by specific task demands.
